# Public Key Encryption with Keyword Search in Cloud: A Survey

**DOI:** 10.3390/e22040421

**Published:** 2020-04-08

**Authors:** Yunhong Zhou, Na Li, Yanmei Tian, Dezhi An, Licheng Wang

**Affiliations:** 1 State Key Laboratory of Networking and Switching Technology, Beijing University of Posts and Telecommunications, Beijing 100876, China; hongdixin@126.com (Y.Z.); 15011177558@163.com (N.L.); tianym0213@163.com (Y.T.); 2 School of Cyber Security, Gansu University of Political Science and Law, Lanzhou 730070, China; adz6199@gsli.edu.cn

**Keywords:** cloud computing, searchable encryption, search query, public key encryption with keyword search, data privacy

## Abstract

With the popularization of cloud computing, many business and individuals prefer to outsource their data to cloud in encrypted form to protect data confidentiality. However, how to search over encrypted data becomes a concern for users. To address this issue, searchable encryption is a novel cryptographic primitive that enables user to search queries over encrypted data stored on an untrusted server while guaranteeing the privacy of the data. Public key encryption with keyword search (PEKS) has received a lot of attention as an important branch. In this paper, we focus on the development of PEKS in cloud by providing a comprehensive research survey. From a technological viewpoint, the existing PEKS schemes can be classified into several variants: PEKS based on public key infrastructure, PEKS based on identity-based encryption, PEKS based on attribute-based encryption, PEKS based on predicate encryption, PEKS based on certificateless encryption, and PEKS supporting proxy re-encryption. Moreover, we propose some potential applications and valuable future research directions in PEKS.

## 1. Introduction

In recent years, with the speedy development of computation and communication, cloud computing [1] is becoming more and more popular, and cloud storage services are becoming more and more mature, such as Baidu Cloud, Amazon simple storage service, Widows Azure, Google Cloud, etc. [2]. As a new type of network storage technology, cloud storage saves user data on the cloud server. Cloud server provider performs corresponding operations on the user data through the online network environment, and charges a fee for use of the hardware resources and service time by the user.

Cloud storage services [3] are widely used in different applications due to its many advantages as follows. It includes data scalability, data accessibility, data shareability, and consistent back up of massive data. Many advantages of cloud storage services improve the quality of the user experience [4] and service, which users are allowed to remotely access data in cloud using any devices from anywhere and at any time instead of having to use fixed machines. Cloud storage is adopted by a large number of individuals and companies in order to reduce the heavy burden of local storage and the management costs.

Despite cloud storage services provide users with a lot of convenience, there are still remaining enormous issues and challenges. When the user outsources their sensitive data to the cloud storage, the transmitted data is vulnerable to intrusion [5,6] by illegal entities especially under critical infrastructures. Meanwhile, the user lost their capabilities to control the data effectively. By accessing the data, the cloud server and the illegal user can try to acquire the information contained in the data, and the privacy security problem of the user is faced with great challenges. In order to protect the security of sensitive data, a straightforward method is used to encrypt the user data before outsourcing it to the cloud [7]. However, when the user wants to search for related files containing a certain keyword, how to process and search on the encrypted data becomes an intractable problem. In the past, there are two methods to solve it. One is to download all encrypted data to the local and then decryption query. This method needs to download a large number of files that are unneeded, which wastes network overhead and requires a lot of computational cost for decryption. This way is not feasible in practice. Another extreme method is user sends the secret key to the cloud server to decrypt the query, but the cloud server is not fully trusted.

In order to better solve the above problems, Goldreich [8] first proposed the ciphertext search mechanism in 1996, but the client and the server needed a large number of interactions, which is not efficient in practical use. In 2000, Song [9] first provided a practical searchable encryption technology, which became a milestone in the development of searchable encryption. Searchable encryption (SE) is a new technology that a user has the capability to selectively search on encrypted data outsourced to the cloud server. From the perspective of cryptography, SE technology mainly includes two types, one is symmetric searchable encryption (SSE), and the other is public key encryption with keyword search (PEKS). At present, an number of SSE schemes [10,11,12,13,14] have been proposed due to high efficiency. However, users have to securely share key for data encryption in SSE, and it is not suitable for multi-user data sharing scenarios. PEKS solved the problem of secret key distribution yielded by SSE. Compared with SSE, PEKS has a broader application prospect. Although there are many survey studies over searchable encryption [15,16,17,18,19], there are few complete survey researches on PEKS. In this article, we seek to complement these existing surveys by presenting a comprehensive study for PEKS schemes.

The remainder of this paper is organized as follows. Section 2 introduces the general framework of PEKS. Section 3 provides a comprehensive taxonomy of existing PEKS schemes in terms of technology view. Section 4 discusses the application area of PEKS. Finally, Section 5 summarizes the paper and provides valuable research directions in this area for future.

## 2. General Framework of PEKS

**Figure 1:**
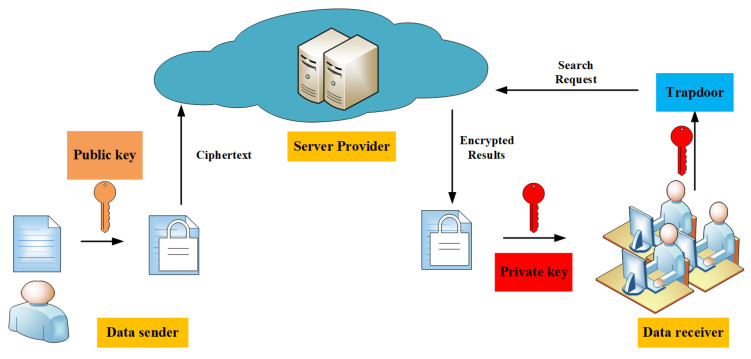
Model of public key encryption with keyword search (PEKS) system.

In 2004, Boneh et al. [20] first proposed the framework of PEKS. PEKS is mainly based on public key encryption algorithms. The PEKS consists of three entities: data sender, data receiver, and cloud server provider. A data sender encrypts their documents and index with the public key and uploads to the remote server provider. A data receiver who has gained corresponding private key can perform the search operation. He generates the trapdoor he wants to search the keyword with the private key and sends it to the server. After receiving trapdoor, the server provider enable test whether a given ciphertext contains the search keyword without knowing the plaintext message of the encrypted data and the keyword. Then, the server provider returns the query results to the data receiver. Finally, the receiver can decrypt the ciphertext which the server sends. PEKS is more suitable for use in some insecure networks. It does not require the encryption party and the decryption party to negotiate the key in advance. Figure 1 shows the model of PEKS system.

### 2.1. Algorithm Description

A general PEKS scheme mainly includes four probabilistic polynomial-time algorithms [20].

- KeyGen(λ): a key generation algorithm run by the **data receiver**. It takes a security parameter λ as input, and outputs public key pk and private key sk.
- PEKS(pk,*W*): an encryption algorithm run by the **data sender**. It takes public key pk and a keyword *W* as inputs, outputs a keyword ciphertext *S* of *W*.
- Trapdoor(sk,*W*): a keyword trapdoor generation run by the **data receiver**. It takes his/her own private key sk and a query keyword *W* as inputs, and outputs the trapdoor $T_{W}$ for the query keyword *W*.
- Test(pk,*S*,$T_{W}$): a test algorithm run by the **server provider**. It takes public key pk, a ciphertext *S* of keyword $W^{\prime }$, and a trapdoor $T_{W}$ of query keyword *W* as inputs, if $W=W^{\prime }$, this algorithm outputs “yes”; otherwise, it outputs “no”.

### 2.2. Security Model

Boneh et al. [20] introduced the first PEKS construction which was based on Boneh and Franklin’s work on IBE [21,22]. They defined the security of PEKS scheme which was indistinguishably secure against an adaptive chosen keyword attack (**IND-CKA**) [20].

We give the following game to definition IND-CKA security between an adversary A and a challenger C.

- **Setup:** The challenger C runs the key generation algorithm to generate public key pk and private key sk. It sends pk to the adversary A and keeps private key sk.
- **Phase 1:** The adversary A can adaptively ask the challenger C for trapdoors corresponding to keywords of its choice.
- **Challenge:** The adversary A sends the challenger C two keywords $W_{0}$, $W_{1}$ on which it wishes to be challenged. Note that $W_{0},W_{1}$ have not been requested before in Phase 1; otherwise, it is a trivial attack that the adversary always wins the game. The challenger C randomly picks a bit b∈{0,1} and gives the adversary A ciphertext $C=PEKS(pk,W_{b})$.
- **Phase 2:** The adversary A can continue to ask for more trapdoors like in Phase 1 for any keyword of his choice except for the $W_{0}$,$W_{1}$.
- **Guess:** The adversary A outputs its guess of $b^{\prime }$ and wins the game if $b=b^{\prime }$.

The advantage of an adversary A in winning the game is

(1) $$Adv_{A}\left(s\right)=\left|Pr\left[b=b^{\prime }\right]-1/2\right|$$

In a short, a PEKS scheme is IND-CKA security if an adversary A has a negligible advantage to win the game.

### 2.3. Attack Model

We investigate the attack model of the PEKS. Although a user can outsource a set of encrypted data to the server provider while maintaining the ability to selectively search over them, most of the existing PEKS schemes are vulnerable to the keyword guessing attack, in addition, there is file injection attack.

#### 2.3.1. Keyword Guessing Attack

Keyword guessing attack (KG) is an important issue for public key searchable encryption. Byun et al. [23] first launched the keyword guessing attack on some PEKS schemes. This attack is originated from the low-entropy property of the keyword space. By performing this attack, an attacker is able to correctly guess the keyword encoded in a given keyword trapdoor. In KG attack, the attacker can be classified into two different types of adversaries, namely, the outside attacker and the inside attacker [24].

- **Outside attacker:** the outside attack is a malicious entity who has no relationship with the server provider and can eavesdrop on a public channel between the server provider and the receiver. Baek et al. [25] first constructed a secure channel free PEKS scheme. If the trapdoor is transmitted over public channel, the outsider attacker can gain the keyword ciphertexts but can not excute the text algorithm.
- **Inside attacker:** the inside attacker usually refers to the malicious server provider. The malicious server can obtain the encrypted keywords from any sender. Meanwhile, it can gain the information about the trapdoor from the receiver. Even worse, if the PEKS scheme is secure channel-free, the malicious server can perform the test algorithm to verify the relation between an encrypted keyword and a trapdoor by using its private key. Obviously it is very difficult to resist inside attacker.

#### 2.3.2. File Injection Attack

File injection attack (FI) is another important issue for public key searchable encryption. Zhang et al. [26] first observed the file injection attack in searchable encryption. A malicious server can obtain query information of the keywords by injecting a set of injected files to the client. File injection attack is devastating for query privacy due to leaking access pattern, it is easy for attacker to recover a significate amount of sensitive data. Therefore, it is imperative to design efficient PEKS schemes to mitigate this critical attack.

### 2.4. Search Functionalities of PEKS

The PEKS is a promising technique in cloud, and it has attracted considerable attentions from cryptographic researchers. In such a case, it is difficult to search over the encrypted data. PEKS is working hard to support information query of various functions in ciphertext like plaintext information retrieval. Up to now, PEKS can support to use various keyword searches, such as single keyword search, conjunctive keyword search, fuzzy keyword search, multi-keyword search, ranking keyword search, verifiable keyword search, similarity keyword search, semantic keyword search, range query, subset query, etc.

## 3. Taxonomy of Existing PEKS Schemes

Since the PEKS was proposed, we have discovered that researchers working in the domain of PEKS use search functionality for the classification of the existing schemes (see, e.g., in [16]). The goal of this section is to give a comprehensive taxonomy of the current PEKS schemes and provide a general overview of the conducted research. In this paper, existing PEKS schemes mainly can be broadly classified into six variants from the technology view, such as PEKS on public key infrastructure, PEKS based on identity-based encryption, PEKS based on attribute-based encryption, PEKS based on predicate encryption, PEKS based on certificateless encryption, and PEKS supporting proxy re-encryption.

### 3.1. PEKS Based on Public Key Infrastructure (PEKS-PKI)

To resolve secret key management and distribution in symmetric searchable encryption, the PEKS was proposed. At current, most PEKS schemes have been established on PKI with the certificate management. The sender has a authority to check the legitimacy of a receiver’s public key and then encrypts the data and keywords under the receiver’s public key; thereafter, it uploads encrypted data to the server provider. The receiver generates the trapdoor under its private key for providing a capability to the server provider to test if a given encrypted data contains the keyword it would like to search. The public key is originated from the third authority namely the public key infrastructure.

#### 3.1.1. PEKS-PKI Research and Progress

Boneh et al. [20] proposed the framework of PEKS based on public key infrastructure using bilinear pairing. Each user in this scheme can be allowed to create searchable content with the receiver’s public key, but only the private key holder can generate a keyword trapdoor to query. Their scheme needs a secure channel to transform the search trapdoor; however, it is expensive to build the secure channel. Baek et al. [25] proposed a PEKS scheme removed the limit for a secure channel. Their construction requires a server public key and private key, and only the server chosen by the sender can search. It was proved security in the random oracle model under the BDH problems. Next, Rhee et al. [27] proposed a new PEKS scheme based on PKI, which enhanced Baek’s model [25] and allowed an attacker to obtain the relation between ciphertexts and a trapdoor.

Park et al. [28] proposed the first PEKS scheme supporting conjunctive keyword search by public key encryption and presented the two constructions based on DBDH problem and DBDHI problem. However, their schemes need amounts of communication and storage overhead. Hwang and Lee [29] improved Park et al. [28] schemes and proposed a new concept called multiuser public key encryption with conjunctive keyword search to save the communication and storage space. Subsequently, Zhang et al. [30] proposed a PEKS scheme supporting conjunctive with subset keyword search and improved Park et al. [28] work that can only support conjunctive keyword search. Lv et al. [31] proposed an expressive and secure PEKS scheme supporting conjunctive, disjunctive and negation search operations based on composite order groups. It was secure in the standard model and can be extended to support range search.

Tang and Chen [32] proposed a PEKS scheme named public key encryption with registered keyword search, which allowed a sender to build searchable content only for the keywords that the sender registered keyword with a receiver first. Their construction was more robust against an offline keyword guessing attack. Hu et al. [33] proposed a decryptable searchable public key encryption with a designated tester construction enhanced security against keyword guessing attacks, and it can decrypt the keyword from keyword ciphertext.

Fang et al. [34] provided a formal model of SCF-PEKS secure against chosen keyword attacks, ciphertext attacks, and keyword guessing attacks. They proposed a secure channel free PEKS scheme without random oracle under the well known assumptions. Next, Shao and Yang [35] enhanced the security model against keyword guessing attacks based on the work of Fang et al. [34] and solved the problem that the attacker is the malicious server. Recently, Lu et al. [24] demonstrated Shao and Yang’s work [35] cannot resist inside keyword guessing attacks and proposed a new improvement of Fang et al. [34] scheme resisted against inside and outside attackers.

Zhang et al. [36] proposed a novel PEKS framework supporting verifiable keyword search and provided two concrete constructions which can maintain the strong security property and have a high efficiency for search over outsourced encrypted data. Huang and Li [37] proposed a public key authenticated encryption with keyword search scheme, in which the data sender not only encrypted keyword but also authenticated it. Their scheme was secure against the inside keyword guessing attack. Recently, Wu et al. [38] proposed a new PEKS construction-based Diffie–Hellman shared secret key to achieve strong security resistance of the file-injected attack and inside keyword guessing attack in existing PEKS systems.

#### 3.1.2. Summary

In Table 1, we compare the several representative PEKS-PKI schemes. Table 2 shows the efficiency of compared PEKS-PKI schemes, and the notations in the Table 3. As usual, the cost of the general cryptographic hash operations are ignored. Although the works in [37,38] fully consider the outside and inside attacks, the method in [37] is more efficient. Therefore, the method in [37] is more suitable for cloud services than other existing PEKS-PKI schemes. The PEKS-PKI scheme needs a certificate to generate a validate public key to prevent public key replacement attacks. However, this will inevitably bring heavy certificate management problems, such as generation, distributions, storage, verification, and revocation.

### 3.2. PEKS Based on Identity-Based Encryption (PEKS-IBE)

**Figure 2:**
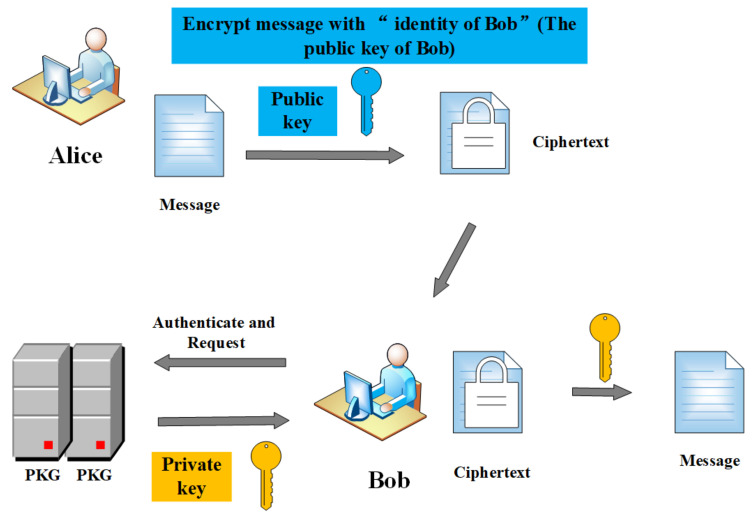
The communication between Alice and Bob.

The identity-based encryption (IBE) was firstly proposed by Shamir [40] in 1984, which simplifies the management of public key and certificate in traditional public key encryption based on PKI. In an identity-based encryption system, a user’s public key can be an arbitrary string such as email address, IP address, telephone number, ID, etc. The private key generation (PKG) can generate the private key according to the user’s authentication and request. Suppose Alice wants to send a message to Bob, the communication step of the two parties using identity-based encryption is shown in Figure 2. In a PEKS-IBE scheme, a data sender uploads ciphertexts to a server provider, then the receiver contacts PKG using its identity to get a corresponding private key and generates a search trapdoor using its private key to send the server provider. Finally, the server provider conducts a keyword search.

#### 3.2.1. PEKS-IBE Research and Progress

Boneh et al. [20] provided the first PEKS scheme based on IBE, in which the keyword acted as the identity. Crescenzo and Saraswat [41] proposed the first PEKS scheme without bilinear maps transformed by the Cocks’ identity-based encryption scheme [42] based on Jacobi symbols and the quadratic residual problem. Their scheme is security in the random oracle model, but it needs secure channels and high storages. Next, Tian et al. [43] improved computation and communication, and proposed an ID-based encryption with keyword search scheme from bilinear pairings which can remove secure channel and be proved secure in random oracle under the appropriate computational assumptions. Subsequently, Camenisch et al. [44] presented an extended notion of PEKS based on blind and anonymous identity-based encryption to improve security. In their scheme, a user is able to obtain a search token from the secret key holder without revealing the keyword.

Abdalla et al. [45] proposed a generic PEKS construction supporting conjunctive keyword from an anonymous IBE scheme and a hierarchical IBE scheme. The server provider performs test algorithm only in a specific time interval, so it cannot use the search trapdoor in the past or future outside the time interval. Next, Khader [46] proposed a PEKS scheme based on k-resilient IBE and presented two construction for conjunctive keyword search and no secure channel to transform trapdoor. Their scheme is secure in the standard oracle under the DDH assumption. However, it needs complex computation and communication space. Subsequently, Wu et al. [47] proposed a novel PEKS secure channel-free scheme with a designated server based on identity-based encryption resisted offline keyword guessing attacks. Recently, Lu et al. [48] pointed out that Wu et al. [47] fails in achieving the ciphertext indistinguishability, and proposed a designated server identity-based encryption scheme supporting conjunctive keyword search removed secure channel and resisted the offline keyword guessing attack.

Said [49] provided a generic transformation from an anonymous IBE to an anonymous (n,t)-IBE in order to implement a novel threshold PEKS deployed on a public key encrypted database. Emura et al. [50] proposed a PEKS scheme supporting keyword revocable based on partially-anonymous identity-based encryption. In their scheme, a keyword trapdoor is generated even if the keyword is revoked, which can resist the security risks caused by the keyword trapdoor. Recently, Wang et al. [51] proposed a secure channel-free identity-based searchable encryption scheme in a peer-to-peer group, which allowed multiple users to share in a peer-to-peer group and search the private data in the cloud.

#### 3.2.2. Summary

In Table 4, we compare several representative PEKS-IBE schemes. Table 5 shows the efficiency of the compared PEKS-IBE schemes, and the notations in the Table 6. Although the methods in [47,48] can resist the outside keyword guessing attack, the method in [47] has more efficiency. Therefore, the method in [47] is more suitable for cloud services than other existing PEKS-IBE schemes. The PEKS-IBE scheme overcomes the certificate management problem based on PKI; however, the current PEKS-IBE schemes have the key escrow issues because a completely trusted private key generator can know all users’ private key.

### 3.3. PEKS Based on Attribute-Based Encryption (PEKS-ABE)

**Figure 3:**
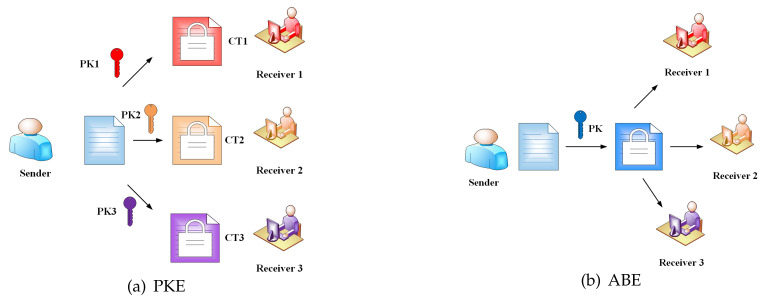
Comparison between public key encryption (PKE) and attribute-based encryption (ABE).

Attribute-based encryption (ABE) was originally proposed by Sahai and Waters [52] in 2005, and is also known as fuzzy identity-based encryption. It regards the identity as a series of attribute sets; attributes are the information elements of the user. We compare traditional public key encryption (PKE) and attribute-based encryption in a multi-user setting. From Figure 3, in PKE, the sender encrypts a document using each receiver’s public key to generate multiple ciphertexts, and each receiver decrypts ciphertexts by using its private key. However, in ABE the sender only needs to formulate an access policy that can be satisfied by multiple users, and then encrypts a document once to generate the only ciphertext. The ciphertext and the user’s private key are associated with the attributes. When user’s attributes match the corresponding access policies, he can decrypt ciphertexts. According to whether the private key or ciphertext is associated with the access control policy, the attribute-based encryption can be further divided into the key-policy attribute-based encryption (KP-ABE) and the ciphertext-policy attribute-based encryption (CP-ABE). In the PEKS-ABE scheme, a data sender allows to grant its search capabilities to receivers by applying an access control policy over the outsourced ciphertexts.

#### 3.3.1. PEKS-ABE Research and Progress

Zhao et al. [53] first considered using the attribute-based signature to realize multi-user keyword search for secure data sharing with fine-grained access control. Han et al. [54] presented a notion based on weak anonymous ABE and considered a transformation ABE into PEKS-ABE. They constructed a concrete PEKS-ABE scheme based on KP-ABE in multi-user setting, but the efficiency was not high. Subsequently, Wang et al. [55] first combined PEKS with CP-ABE and proposed a ciphertext-policy attributed-based encryption scheme to support keyword search functionality. This scheme allows the data sender to control his data access policy, and only legitimate data receivers who meet the policy can retrieve the keyword and decrypt the ciphertext.

Zheng et al. [56] introduced a verifiable attribute-based keyword search scheme, in which the data user can control the search and use of the encrypted data and verify whether the server provider conducts correct keyword search. However, their scheme needs a secure channel and the verification cost is expensive. Liu et al. [57] improved Zheng et al.’s [56] work and proposed a relatively efficient PEKS-ABE scheme based on key policy attribute keyword search removed a secure channel. Subsequently, Li et al. [58] devised a fine-grained access control system to decrease the computation resources in PEKS-ABE, and the server provider can perform partial decryption operations without learning any information related to plaintext.

Yang et al. [59] provided a PEKS-ABE scheme based on bilinear pairing to support fine-grained access control and semantic keyword search in the multi-user settings that enable convenient user revocation mechanism, but it cannot consider traceability. Next, Ning et al. [60] proposed a PEKS-ABE scheme based on CP-ABE supporting traceability by embedding their identity information in the secret keys to prevent dishonest data users from leaking their secret keys to others. Subsequently, Sun et al. [61] proposed a PEKS-ABE scheme supporting efficient user revocation allowed multiple senders to encrypt and outsource the data to the server provider independently. The receivers are able to generate their own search abilities without relying on an trusted authority that is always online. Recently, Zhu et al. [62] proposed a PEKS-ABE scheme based on ciphertext policy attribute-based encryption in order to support with access control over ciphertext and fuzzy keyword search.

Miao et al. [63] proposed a basic attribute-based keyword search over hierarchical data scheme by using CP-ABE technique. Because the basic scheme cannot satisfy the desirable requirements in cloud, they provided two improved schemes supporting multi-keyword search and user revocation. However, they can not consider attack models. Next, Cao et al. [64] enhanced the security that can resist the collision attacks of the server provider and the data user. They proposed a PKES-ABE scheme based on blinded CP-ABE in cloud, which blinded the access attributes of the users. The server provider not only performs the keyword search but also conducts pre-decryption operation. In shared multi-owner setting, Miao et al. [65] recently proposed a privacy preserving PEKS-ABE system by using CP-ABE technology with hidden access policy achieved selective security in the generic bilinear group model, and it can resist the offline keyword guessing attack.

#### 3.3.2. Summary

In Table 7, we compare the several representative PEKS-ABE schemes. Table 8 shows the efficiency of compared PEKS-ABE schemes, and the notations in the Table 9. The method in [64] has relatively high efficiency, but it cannot resist the keyword guessing attack. Therefore, the method in [65] is more suitable for cloud services than other existing PEKS-ABE schemes. The attribute-based encryption method is widely adopted in PEKS due to its efficient data sharing and searching ability. However, the PEKS-ABE scheme also brings the key escrow problem simultaneously because all users’ private keys are known by the PKG.

### 3.4. PEKS Based on Predicate Encryption (PEKS-PE)

The notion of predicate encryption was first proposed by Katz et al. [66] in 2008. Predicate encryption is a new public key encryption allowing users to search on ciphertext without a private key corresponding to a public key, and it can be achievable to fine-grained access control on encrypted data. Secret keys are associated with predicates and ciphertexts are associated with attributes in the predicate encryption system. According to access control, predicate encryption can achieve high flexibility supporting attribute-hiding and payload-hiding. Therefore, predicate encryption might be applied to search over encrypted data. In a PEKS-PE scheme, the server provider can perform the test algorithm to match ciphertexts with the trapdoor supplied on particular predicates. Finally, the server provider returns the query results to the receiver without revealing any information to the server. Corresponding to a predicate, the receiver owning the secret key can decrypt the resulting ciphertexts associated with attributes and recover the message.

#### 3.4.1. PEKS-PE Research and Progress

Blundo et al. [67] proposed a predicate encryption scheme with partial public key by the need for predicate privacy in PEKS. They defined token security to ensure the privacy of attributes from a token. To reduce the communication cost between the receiver and sender, Zhu et al. [68] provided a extend PEKS scheme based on predicate encryption to support predicate privacy, and it based on the idea of randomization without requiring interaction between the receiver and sender. Next, Katz et al. [69] proposed a PEKS-PE scheme supporting disjunctive keyword search, and provided the approach of converting a predicate encryption scheme into a PEKS. Zhang et al. [70] proposed a PEKS-PE scheme, which can not only support disjunctive keyword search, but also conjunctive keyword search over encrypted data. Kim et al. [71] proposed an efficient predicate encryption with constant pairing computations supporting the evaluations of polynomials, disjunctions, conjunctions, CNF formulas, and threshold. Recently, Zhang et al. [72] proposed an efficient PEKS-PE scheme to support conjunctive and disjunctive keyword search, and it needs less time and storage consumption.

Gay et al. [73] proposed a lattice-based PEKS-PE scheme for multidimensional range and multidimensional subset queries, and it was selectively secure and weakly attribute-hiding under the standard learning with errors assumption. Recently, Zhang et al. [74] constructed a PEKS to support semantic multi-keyword search through applying an efficient predicate encryption. In their scheme, the semantic index and query keyword set can be converted into an attribute and a predict vector by utilizing a keyword conversion method.

#### 3.4.2. Summary

In Table 10, we compare the several representative PEKS-PE schemes. Table 11 shows the efficiency of compared PEKS-PE schemes, and the notations in the Table 12. The scheme of Zhang et al. [74] can resist the keyword guessing attack and has relatively strong efficiency. It is more applicable to the cloud environment. Predicate encryption is a new paradigm that covering identity-based encryption, attribute-based encryption, and hidden vector encryption. Attribute information is public in attribute-based encryption, whereas attribute information is hidden in predicate encryption.

### 3.5. PEKS Based on Certificateless Encryption (PEKS-CLE)

AI-Riyami and Paterson [75] first proposed certificateless encryption in 2003, which is a new type of public key cryptosystem based on the identity-based public key cryptosystem. The private key in certificateless public key encryption is no longer independently generated by the PKG, but is jointly generated by the PKG and the user. In a PEKS-CLE scheme, a data sender encrypts both a keyword and data using a receiver’s public key and identity, then it sends encrypted data to the server provider. The receiver first obtains a partial private key from the PKG. A complete private key combines a partial private key and a secret value chosen by the receiver. Thereafter, the receiver generates the search trapdoor using its complete private key to send the server provider in order to conduct search keyword. Certificateless encryption overcomes the problem of key escrow in identity-based encryption because the secret key is only known by the receiver and the PKG does not know it.

#### 3.5.1. PEKS-CLE Research and Progress

Peng et al. [76] first introduced certificateless encryption into PEKS and constructed an secure channel free PEKS-CLE scheme, and it was secure against chosen keyword attack and keyword guessing attack. Subsequently, Wu et al. [77] demonstrated that the certificateless searchable public key encryption scheme of Peng et al. [76] cannot resist a malicious PKG attack and an offline keyword guessing attack.

Zheng et al. [78] integrated the certificateless cryptography with keyword search and proposed a PEKS-CLE scheme that was provably secure in the standard model under the decisional linear assumption, but it cannot consider attack model. Next, Ma et al. [79] proposed a new PEKS-CLE scheme supporting multiple keyword search for industrial Internet of Things deployment removed secure channel, and it was proved security in the random oracle model against a malicious PKG attack and public key replacement attack. Subsequently, Ma et al. [80] proposed an efficient PEKS-CLE scheme for mobile health care system to remove the key management problem and key escrow problem, and it can resist the chosen keyword and keyword guessing attack in the random oracle model.

Islam et al. [81] proposed a PEKS-CLE with designated server scheme that was secure under the bilinear Diffie–Hellman assumption and computational Diffie–Hellman assumption. Next, Wu et al. [82] enhanced the security resisted various types of attacks, and they constructed a new certificateless public key authenticated encryption with keyword search utilizing designated tester for cloud-assisted mIoT. Recently, Lu et al. [83] proposed a pairing-free PEKS-CLE scheme to improve the efficiency problems caused by the use of bilinear pairing, and this scheme was formally proved its security under the complexity assumption of the CDH problem in the random oracle model.

#### 3.5.2. Summary

In Table 13, we compare the several representative PEKS-CLE schemes. Table 14 shows the efficiency of compared PEKS-CLE schemes, and the notations in the Table 15. The method in [83] has greater efficiency but it cannot resist the keyword guessing attack; the method in [82] can resist all kinds of attacks. Therefore, the method in [82] is more suitable for cloud services than other existing PEKS-CLE schemes. The PEKS-CLE scheme not only overcomes certificate management problems based on PKI, but also resolves the key escrow problem based on IBE and ABE. In other words, certificateless encryption lies between conventional public key encryption and identity-based encryption, but preserves the certificateless advantages.

### 3.6. PEKS Supporting Proxy Re-Encryption (PEKS-PRE)

**Figure 4:**
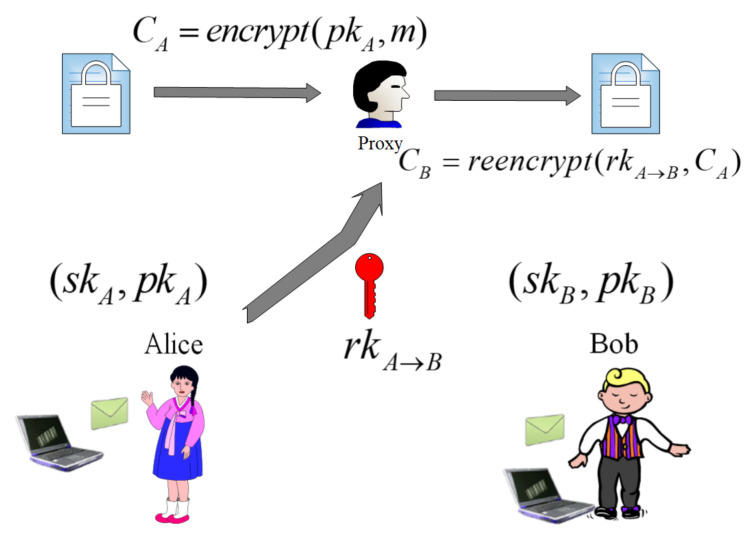
PRE model.

Proxy re-encryption (PRE) was first proposed by Blaze, Bleumer, and Strauss [84] in Eurocrypt’98. In a proxy re-encryption mechanism, a semi-trusted third party called a proxy is responsible for the ciphertext conversion. In some situations, a sender that acts as a delegator is allowed to delegate their search right to a delegatee through re-encrypting the ciphertext, without revealing his own private key. The ciphertext conversion requires to generate re-encryption key using delegator’s private key and delegatee’s public key. In this process, the proxy cannot obtain relevant plaintext information. Suppose Alice is a delegator and Bob is a delegatee, the proxy re-encryption model is shown in Figure 4. PEKS-PRE is a cryptographic primitive for searching on encrypted information without decrypting it while supporting a proxy re-encryption system. The server provider plays role in a proxy that could convert the encrypted data into a re-encrypted ciphertext searched by the delegatee. A data sender allows the authorization of the search ability to the other receiver. The proxy cloud server is given a trapdoor which it can use to test whether or not a ciphertext contains a keyword without knowing anything else about the contents of data and keyword.

#### 3.6.1. PEKS-PRE Research and Progress

Shao et al. [85] first integrated proxy-encryption and PEKS, which a data user allowed to delegate keyword search ability to another user. They provided a bidirectional PEKS-PRE scheme that was secure in the random oracle model. Yao et al. [86] proposed a novel PEKS-PRE scheme with a designated tester that extended original PEKS model by adding algorithms of re-encrytion key generation and re-encryption of keyword ciphertext to satisfy the requirements of applications. Subsequently, Zhong et at. [87] proposed PEKS from anonymous conditional proxy re-encryption ensured the privacy and security of ciphertext.

Wang et al. [88] proposed a PEKS-PRE scheme supporting conjunctive keyword search constrained single-hop unidirectional proxy re-encryption under the bilinear pairing, and it was secure in the random oracle model. Guo et al. [89] enhanced the security, and they proposed a new searchable bidirectional proxy re-encryption with a designer server without resorting to random oracle model supported verifiable correctness of the keyword results. Next, Yang et al. [90] proposed a PEKS-PRE scheme with a designated tester for electronic health record system supporting secure conjunctive keyword search. The patients enable delegate partial access rights to others to operate search functions over their records in a limited time period. Recently, Yang et al. [91] proposed a novel semantic keyword searchable proxy re-encryption scheme resisted quantum attack, and this scheme was proven secure in the standard model under the learning with errors hardness problem.

Shi et al. [92] combined attribute-based encryption and proxy re-encryption with keyword search and provided two construction schemes based on KP-ABE and CP-ABE. The data user is allowed to delegate the keyword search ability to multiple users. Next, Chen et al. [93] proposed a restricted proxy re-encryption with keyword search scheme for fine-grained data access control to restrict the capability of the proxy cloud server. Subsequently, Hong et al. [94] proposed an attribute-based proxy re-encryption scheme with keyword search, and it fully took advantage of the attribute-based re-encryption and keyword search supporting flexible data access control among users in data sharing scenario. Recently, Chen et al. [95] also combined proxy re-encryption with the attribute-keyword search and provided an attribute-based keyword search with proxy re-encryption scheme that achieved the functions of the data search and fine-grained access control.

#### 3.6.2. Summary

In Table 16, we compare the several representative PEKS-PRE schemes. Table 17 shows the efficiency of the compared PEKS-PRE schemes, and the notations in the Table 18. The methods in [89,90] can resist the keyword guessing attack. However, the method in [90] has more efficiency applying to conjunctive keyword search in cloud. Therefore, the method in [90] is more suitable for cloud services than other existing PEKS-PRE schemes. Proxy re-encryption provides convenience on encrypted data search improved the functions of revocation, update and deletion of ciphertext data.

## 4. Application Area

PEKS is a hot research topic of searchable encryption that has great potential for use in many applications where confidential data is outsourced to the third party server provider without affecting the usage of the data stored in the cloud. This section mainly provides some applications of PEKS, such as e-mail routing, health care, and smart grid etc.

### 4.1. E-mail Routing

Boneh et al. [20] proposed the first PEKS scheme in the e-mail routing scenario. Suppose Bob sends an e-mail containing certain keyword to Alice through an untrusted mail server. It is required that the server cannot obtain the email content and related keyword information, but it needs to route the email to the corresponding terminal device of Alice according to the keyword information. For example, when the keyword information is “urgent”, the server forwards the message to Alice’s mobile phone. When the keyword information is “lunch”, the server forwards the message to Alice’s laptop. Alice is able to read the message on whatever device he/she wants. To protect the privacy of emails, the sender encrypts e-mail with receiver’s public key. PEKS might be achievable to search over encrypted email.

### 4.2. Health Care

For medical practices, an increasing number of health care providers tend to deploy the electronic medical record storage and application services into a third party cloud in order to reduce the cost of huge data storage and maintenance. When medical data are stored on a cloud server that is not fully trusted, patient privacy and security becomes critical issues. To protect the confidentiality of sensitive data, the health care providers prefers to encrypt the medical data before uploading to the cloud. In electronic health care systems, the details of a patient are utilized by the doctors for diagnosis of the disease shared with other doctors. PEKS can provide searching the encrypted data and sharing with authorized user, there are many researches for health care [96,97,98,99,100].

### 4.3. Smart Grid

In the power system world, smart grid is a new revolution using the IoT technology that it increasingly attracts the attention of many organizations. The smart grid system can not only measure and collect energy usage data through sensors and smart meters, but also store and utilize energy usage data through a powerful cloud computing platform. As the power information uploads to the cloud, security, and privacy of these data become vital. A professional controller prefers to encrypt the sensitive information before outsourcing to cloud, which makes PEKS applicable to smart grid system [101,102,103,104].

## 5. Conclusions and Future Directions

PEKS has been researched for years and has made great progress in recent years. Nevertheless, there are still many drawbacks or problems to be resolved. Various PEKS researches focus on three main influencing factors, namely, query expressiveness, efficiency, and security.

- **Query expressiveness.** The existing PEKS schemes have been improved in order to make them more practical for deployment in different application devices, which not only can support single keyword search but also support conjunctive keyword search, fuzzy keyword search, semantic keyword search, rank search, range search, and subset search. However, this needs to be achieved at the cost of efficiency and security.
- **Efficiency.** A large number of existing PEKS schemes are constructed based on bilinear pairings that are inefficient due to a lot of computational overhead. How to construct a scheme without bilinear map to improve the efficiency of PEKS becomes an important issue that needs to be solved. At the same time, existing multi-user PEKS schemes are not practical in real-world applications and cannot scale well for large constructions. Thus, one of the goal of PEKS schemes should reduce the computational complexity.
- **Security.** Most of existing PEKS schemes are proved security under the random model. However, the random model has its limitations. Security can be proven under the random model, but it may not be secure in practical applications. Although there are some schemes that can be proved security under the standard model, they are usually inefficient and require a large amount of computation costs and space storage. In addition, most PEKS schemes are vulnerable to keyword guessing attacks and file injection attacks.

The research of PEKS still requires to concentrate on improving the query expressiveness and the trade-off between the efficiency and security at the same time. With recent research progress, the future development direction of public key searchable encryption includes the following aspects.

- **Multi-Source Data.** With the rapid development of the social network, multimedia information has grown at an explosive speed, especially multimedia information represented by videos and images. To protect the data privacy, the data information containing sensitive content needs to be encrypted before uploading to the cloud. However, how to query the required video or image on encrypted data has become an intractable problem. In the future, we can consider how to apply PEKS to deal with such problems.
- **Lattice-Based PEKS.** With the growth of the quantum computing, traditional cryptographic algorithms based on hardness assumption will face a huge challenge that can easily be attacked by the quantum computer. Consequently, it is necessary to design a cryptographic algorithm that can resist quantum attack. Lattice-based cryptosystems are becoming increasingly popular in the post-quantum algorithms due to their efficiency and conceptual simplicity. Some researches have evolved [105,106,107,108], and it is urgent to construct an efficient and secure lattice-based PEKS scheme in the future.
- **Blockchain-Based PEKS.** As an emerging integrated technology, the blockchain plays an important role in new technological innovation, and nowadays it is increasing attracting attention from all walks of life. Specially, the data requires to be encrypt so as to achieve confidentiality before storing on the blockchain. However, it becomes difficult to conduct keyword search over the blockchain. PEKS can help the effective utilization of the encrypted data while protecting the data privacy. Several studies [109,110,111] have been proposed for PEKS based on blockchain. For future progress in this field, more research efforts are required.

## Figures and Tables

**Table 1:** Comparison of several PEKS-PKI schemes.

Scheme	Search Functionality	Security				Attack Model		
		Definition	Assumption	ROM	SCF	OKG	IKG	FI
Boneh et al. [20]	Single	IND-CKA	BDH	✓				
Park et al. [28]-I	Conjunctive	IND-CKA	DBDH	✓				
Park et al. [28]-II	Conjunctive	IND-CKA	DBDHI	✓				
Hwang et al. [29]	Conjunctive	IND-CKA	DLDH	✓				
Baek et al. [25]	Single	IND-CKA	BDH	✓	✓			
Rhee et al. [27]	Single	IND-CKA	BDH,BDHI	✓	✓			
Tang et al. [32]	Single	IND-CKA	DBDH	✓	✓	✓		
Zhang et al. [30]	Conjunctive, subset	TU,AC	DDHI	-		✓		
Hu et al. [33]	Single	IND-CKA	DLP,HDH		✓	✓		
Shao et al. [35]	Single	IND-KGAs	-	-	✓	✓		
Huang et al. [37]	Single	SS	DBDH,mDLIN	✓		✓	✓	✓
Wu et al. [38]	Single	IND-CKA	DBDH,CDH			✓	✓	✓

ROM denotes random oracle model. (ROM [39] is an ideal oracle for modelling a cryptographic hash function.) SCF denotes secure channel free. OKG denotes outside keyword guessing attack. IKG denotes inside keyword guessing attack and FI denotes file injection attack. BDH refers to Bilinear Diffie–Hellman assumption. DBDH refers to Decisional Bilinear Diffie–Hellman assumption. DBDHI refers to Decisional Bilinear Diffie–Hellman Inversion assumption. DLDH refers to Decision Linear Diffie–Hellman assumption. BDHI refers to Bilinear Diffie–Hellman Inversion assumption. DDHI refers to Decisional Diffie–Hellman Inversion assumption. DLP refers to Discrete Logarithm Problem. HDH refers to Hash Diffie–Hellman assumption. mDLIN refers to modified Decision Linear assumption. CDH refers to Computational Diffie–Hellman assumption. We write SS for sematic security, TU for trapdoor unforgetable, AC for anonymous of the ciphertext, and IND-KGAs for IND-KGA-server.

**Table 2:** Efficiency of the compared PEKS-PKI schemes.

Scheme	Computation Cost			Communication Cost	
	Encrypt	Trapdoor	Test	Ciphertext Size	Trapdoor Size
Boneh et al. [20]	$2T_{exp}+T_{\hat{e}}$	$T_{exp}$	$T_{\hat{e}}$	$log|G_{1}\left|+|\lambda \right|$	$log|G_{1}|$
Park et al. [28]-I	$\left(l+2\right)T_{exp}+lT_{\hat{e}}$	$T_{exp}$	$T_{exp}+T_{\hat{e}}$	$2log|G_{1}\left|+llog\right|G_{t}|$	$log|G_{1}\left|+log\right|Z_{p}|$
Park et al. [28]-II	$\left(3l+2\right)T_{exp}$	$2T_{exp}$	$T_{exp}+2T_{\hat{e}}$	$\left(2l+3\right)log|G_{1}|$	$2log|G_{1}\left|+log\right|Z_{p}|$
Hwang et al. [29]	$\left(2l+2\right)T_{exp}$	$3T_{exp}$	$3T_{\hat{e}}$	$\left(l+2\right)log|G_{1}|$	$3log|G_{1}|$
Baek et al. [25]	$2T_{exp}+2T_{\hat{e}}$	$T_{exp}$	$T_{exp}+T_{\hat{e}}$	$log|G_{1}\left|+|\lambda \right|$	$log|G_{1}|$
Rhee et al. [27]	$2T_{exp}+9T_{\hat{e}}$	$T_{exp}$	$T_{exp}+T_{\hat{e}}$	$log|G_{1}\left|+|\lambda \right|$	$log|G_{1}|$
Tang et al. [32]	$3T_{exp}+T_{\hat{e}}$	$T_{exp}$	$T_{\hat{e}}$	$log|G_{1}\left|+log\right|G_{t}|$	$log|G_{1}|$
Zhang et al. [30]	$\left(2l+2\right)T_{exp}+T_{\hat{e}}$	$3lT_{exp}$	$\left(2+2l\right)T_{exp}+\left(2l+1\right)T_{\hat{e}}$	$\left(l+1\right)log|G_{1}|+$ $\left(l+2\right)log|G_{t}\left|+log\right|Z_{p}|$	$\left(l+2\right)log|G_{1}\left|+log\right|G_{t}|$
Hu et al. [33]	$2T_{exp}+3T_{\hat{e}}$	$3T_{exp}$	$2T_{exp}+T_{\hat{e}}$	$log|G_{1}\left|+log\right|Z_{p}|$	$2log|G_{t}|$
Shao et al. [35]	$9T_{exp}+3T_{\hat{e}}$	$2T_{exp}$	$5T_{exp}+4T_{\hat{e}}$	$5log|G_{1}\left|+3log\right|G_{t}|$	$3log|G_{1}|$
Huang et al. [37]	$3T_{exp}$	$T_{exp}+T_{\hat{e}}$	$2T_{\hat{e}}$	$2log|G_{1}|$	$log|G_{t}|$
Wu et al. [38]	$5T_{exp}+T_{\hat{e}}$	$5T_{exp}$	$6T_{exp}+3T_{\hat{e}}$	$2log|G_{1}\left|+log\right|G_{t}|$	$2log|G_{1}|$

**Table 3:** Notations for PEKS-PKI schemes.

Notation	Description
$T_{exp}$	The time of a modular exponentiation
$T_{\hat{e}}$	The time of a bilinear pairing
$|G_{1}|$	The number of elements in $G_{1}$
$|G_{t}|$	The number of elements in $G_{t}$
$|Z_{p}|$	The number of elements in $Z_{p}$
λ	The security parameter
|λ|	The bit length of security parameter
*l*	The number of the keywords

**Table 4:** Comparison of several PEKS-IBE schemes.

Scheme	Search Functionality	Security				Attack Model		
		Definition	Assumption	ROM	SCF	OKG	IKG	FI
Boneh et al. [20]	Single	IND-CKA	BDH	✓				
Khader et al. [46]	conjunctive	IND-CKA	DDH		✓			
Crescenzo et al. [41]	Single	IND-CKA	QIP	✓				
Tian et al. [43]	Single	IND-CKA	DLP	✓	✓			
Wu et al. [47]	Conjunctive	IND-CKA	BDH,CDH	✓	✓	✓		
Wang et al. [51]	Multi-user	IND-CKA	DBDH	✓	✓	✓		
Lu et al. [48]	Conjunctive	IND-CKA	DBDH,CDH	✓	✓	✓		

BDH refers to Bilinear Diffie–Hellman assumption. DBDH refers to Decisional Bilinear Diffie–Hellman assumption. DDH refers to Decisional Diffie–Hellman assumption. QIP refers to Quadratic Indistinguishability Problem. DLP refers to Discrete Logarithm Problem. CDH refers to Computational Diffie–Hellman assumption.

**Table 5:** Efficiency of the compared PEKS-IBE schemes.

Scheme	Computation Cost			Communication Cost	
	Encrypt	Trapdoor	Test	Ciphertext Size	Trapdoor Size
Boneh et al. [20]	$2T_{exp}+T_{\hat{e}}$	$T_{exp}$	$T_{\hat{e}}$	$log|G_{1}\left|+|\lambda \right|$	$log|G_{1}|$
Khader et al. [46]	$\left(3l\lambda +3l+3\right)T_{exp}$	-	$5T_{exp}$	$\left(3+2l\right)log|G_{1}|$	$\left(4+l\right)log|Z_{p}|$
Crescenzo et al. [41]	4λJ	$4\lambda T_{exp}$	$4\lambda (J+T_{exp})$	$4|\lambda |log|Z_{p}|$	$4|\lambda |log|Z_{p}|$
Tian et al. [43]	$3T_{exp}$	$T_{exp}$	$2T_{\hat{e}}$	$3log|G_{1}|$	$log|G_{1}|$
Wu et al. [47]	$\left(l+2\right)T_{exp}+T_{\hat{e}}$	$2T_{exp}$	$2T_{exp}+2T_{\hat{e}}$	$\left(l+1\right)log|G_{1}\left|+|\lambda \right|$	$2log|G_{1}|$
Wang et al. [51]	$\left(5+n^{2}\right)T_{exp}+T_{\hat{e}}$	$5T_{exp}+T_{\hat{e}}$	$nT_{exp}+4T_{\hat{e}}$	$\left(n+3\right)log|G_{1}\left|+log\right|G_{t}|$	$3log|G_{1}|$
Lu et al. [48]	$\left(l+4\right)T_{exp}+T_{\hat{e}}$	$3T_{exp}+T_{\hat{e}}$	$3T_{exp}+3T_{\hat{e}}$	$\left(l+3\right)log|G_{1}\left|+2log\right|Z_{p}|$	$2log|G_{1}\left|+log\right|Z_{p}|$

**Table 6:** Notations for PEKS-IBE schemes.

Notation	Description
$T_{exp}$	The time of a modular exponentiation
$T_{\hat{e}}$	The time of a bilinear pairing
$|G_{1}|$	The number of elements in $G_{1}$
$|G_{t}|$	The number of elements in $G_{t}$
$|Z_{p}|$	The number of elements in $Z_{p}$
λ	The security parameter
|λ|	The bit length of security parameter
*l*	The number of the keywords
*J*	The Jacobi symbol
*n*	The number of the users share the data

**Table 7:** Comparison of several PEKS-ABE schemes.

Scheme	Search Functionality	Security				Attack Model		
		Definition	Assumption	ROM	SCF	OKG	IKG	FI
Wang et al. [55]	Single	SeS	q-DBDH	-		✓	✓	
Zheng et al. [56]-I	Verifiable	IND-CKA	DLIN	✓				
Zheng et al. [56]-II	Verifiable	IND-CKA	DLIN					
Sun et al. [61]	Verifiable	IND-CKA	DBDH					
Li et al. [58]	Single	CPA	DBDH	✓			✓	
Miao et al. [63]	Multi-keyword	IND-CKA	DBDH	✓				
Cao et al. [64]	Single	IND-CKA	BDH	✓				
Miao et al. [65]	Single	SeS	DBDH			✓		

BDH refers to Bilinear Diffie–Hellman assumption. DBDH refers to Decisional Bilinear Diffie–Hellman assumption. q-DBDH refers to q-parallel Decisional Bilinear Diffie–Hellman assumption. DLIN refers to Decisional Linear assumption. We write SeS for selective security and CPA for choose plaintext attack.

**Table 8:** Efficiency of the compared PEKS-ABE schemes.

Scheme	Computation Cost			Communication Cost	
	Encrypt	Trapdoor	Test	Ciphertext Size	Trapdoor Size
Wang et al. [55]	$\left(3N+2\right)T_{exp}+T_{\hat{e}}$	$\left(S+2\right)T_{exp}$	$NT_{exp}+\left(2N+1\right)T_{\hat{e}}$	$\left(2N+1\right)log|G_{1}\left|+log\right|G_{t}|$	$\left(S+2\right)log|G_{1}|$
Zheng et al. [56]-I	$\left(S+4\right)T_{exp}$	$\left(2N+2\right)T_{exp}$	$ST_{exp}+\left(2S+2\right)T_{\hat{e}}$	$\left(S+3\right)log|G_{1}|$	$\left(2N+2\right)log|G_{1}|$
Zheng et al. [56]-II	$\left(2N+4\right)T_{exp}$	$\left(2S+4\right)T_{exp}$	$NT_{exp}+\left(2N+3\right)T_{\hat{e}}$	$\left(2N+3\right)log|G_{1}|$	$\left(2S+3\right)log|G_{1}|$
Li et al. [58]	$\left(2+S\right)T_{exp}+T_{\hat{e}}$	$\left(3+3N\right)T_{exp}$	$2T_{exp}$	$\left(S+1\right)log|G_{1}\left|+log\right|G_{t}|$	$4log|G_{1}|$
Sun et al. [61]	$\left(N+2\right)T_{exp}$	$\left(2N+1\right)T_{exp}$	$T_{exp}+\left(N+1\right)T_{\hat{e}}$	$\left(2N+1\right)log|G_{1}\left|+log\right|G_{t}|$	$\left(2N+1\right)log|G_{1}\left|+log\right|Z_{p}|$
Miao et al. [63]	$\left(2N+1\right)T_{exp}$	$\left(2S+4\right)T_{exp}$	$T_{exp}+\left(2S+3\right)T_{\hat{e}}$	$log|G_{1}\left|+2log\right|G_{t}|$	$\left(2S+3\right)log|G_{1}|$
Cao et al. [64]	$\left(3+S\right)T_{exp}+T_{\hat{e}}$	$2T_{exp}$	$4T_{\hat{e}}$	$\left(3+N\right)log|G_{1}|$	$2log|G_{1}|$
Miao et al. [65]	$\left(2+2N\right)T_{exp}+T_{\hat{e}}$	$\left(2N+1\right)T_{exp}$	$T_{exp}+\left(2N+1\right)T_{\hat{e}}$	$\left(N+1\right)log|G_{1}\left|+log\right|G_{t}|$ $+log|Z_{p}|$	$\left(2N+1\right)log|G_{1}\left|+log\right|Z_{p}|$

**Table 9:** Notations for PEKS-ABE schemes.

Notation	Description
$T_{exp}$	The time of a modular exponentiation
$T_{\hat{e}}$	The time of a bilinear pairing
$|G_{1}|$	The number of elements in $G_{1}$
$|G_{t}|$	The number of elements in $G_{t}$
$|Z_{p}|$	The number of elements in $Z_{p}$
*S*	The number of a data user’s attribute
*N*	The number of attributes that are involved in a data owner’s access control policy

**Table 10:** Comparison of several PEKS-PE schemes.

Scheme	Search Functionality	Security				Attack Model		
		Definition	Assumption	ROM	SCF	OKG	IKG	FI
Zhu et al. [68]	Single	PP,SP	ECDLP	-				
Zhang et al. [70]	Disjunctive,conjunctive	CPA	-	-				
Zhang et al. [74]	Semantic	CPA,IND-CKA	-	-		✓		
Zhang et al. [72]	Conjunctive,disjunctive	IND-CKA	BDHI,DLIN	✓				

ECDLP refers to Elliptic Curve Discrete Logarithm Problem, BDHI refers to Bilinear Diffie–Hellman Inversion assumption, DLIN refers to Decision Linear assumption. We write PP for predicate privacy, SP for statistics privacy and CPA for choose plaintext attack.

**Table 11:** Efficiency of the compared PEKS-PE schemes.

Scheme	Computation Cost			Communication Cost	
	Encrypt	Trapdoor	Test	Ciphertext Size	Trapdoor Size
Zhu et al. [68]	$2T_{exp}+T_{\hat{e}}$	$T_{exp}$	$T_{\hat{e}}$	$log|G_{1}\left|+|\lambda \right|$	$log|G_{1}|$
Zhang et al. [70]	$\left(4l^{2}+3l\right)T_{exp}$	$\left(4l+2\right)T_{exp}$	$2l\left(2l+1\right)T_{\hat{e}}$	$\left(4l^{2}+2l\right)log\left|G_{1}\right|$ $+log|G_{t}|$	$\left(4l+2\right)log|G_{1}|$
Zhang et al. [74]	$\left(2l+4\right)T_{exp}$	$\left(l+4\right)T_{exp}$	$\left(l+1\right)T_{exp}+3T_{\hat{e}}$	$\left(l+3\right)log|G_{1}|$	$3log|G_{t}\left|+\left(l+1\right)log\right|Z_{p}|$
Zhang et al. [72]	$\left(3l^{2}+4l+1\right)T_{exp}$	$\left(2l+2\right)T_{exp}$	$2l\left(l+1\right)T_{\hat{e}}$	$\left(2l^{2}+4l\right)log|G_{1}\left|+log\right|G_{t}|$	$\left(2l+2\right)log|G_{1}\left|+log\right|Z_{p}|$

**Table 12:** Notations for PEKS-PE schemes.

Notation	Description
$T_{exp}$	The time of a modular exponentiation
$T_{\hat{e}}$	The time of a bilinear pairing
$|G_{1}|$	The number of elements in $G_{1}$
$|G_{t}|$	The number of elements in $G_{t}$
$|Z_{p}|$	The number of elements in $Z_{p}$
*l*	The number of the keywords
|λ|	The bit length of security parameter

**Table 13:** Comparison of several PEKS-CLE schemes.

Scheme	Search Functionality	Security				Attack Model		
		Definition	Assumption	ROM	SCF	OKG	IKG	FI
Peng et al. [76]	Single	IND-CKA	BDH	✓	✓	✓		
Zheng et al. [78]	Single	CI	DLIN					
Islam et al. [81]	Single	CI,DI	CDH,BDH	-	✓	✓		
Ma et al. [80]	Single	IND-CKA	BDH	✓		✓		
Wu et al. [82]	Single	SS	CBDH	✓	✓	✓	✓	
Lu et al. [83]	Single	IND-CKA	CDH	✓				

BDH refers to Bilinear Diffie–Hellman assumption. DLIN refers to Decisional Linear assumption. CDH refers to Computational Diffie–Hellman assumption. CBDH refers to Computational Bilinear Diffie–Hellman assumption. We write CI for ciphertext indistinguishability, DI for trapdoor indistinguishability, and SS for semantically secure.

**Table 14:** Efficiency of the compared PEKS-CLE schemes.

Scheme	Computation Cost			Communication Cost	
	Encrypt	Trapdoor	Test	Ciphertext Size	Trapdoor Size
Peng et al. [76]	$4T_{exp}+7T_{\hat{e}}$	$4T_{exp}$	$2T_{exp}+T_{\hat{e}}$	$log|G_{1}\left|+log\right|Z_{p}|$	$3log|G_{1}|$
Zheng et al. [78]	$5T_{exp}$	$8T_{exp}$	$4T_{\hat{e}}$	$4log|G_{1}|$	$4log|G_{1}|$
Islam et al. [81]	$5T_{exp}$	$T_{exp}$	$3T_{exp}+2T_{\hat{e}}$	$3log|G_{1}|$	$log|G_{1}|$
Ma et al. [80]	$5T_{exp}+3T_{\hat{e}}$	$2T_{exp}$	$3T_{exp}+T_{\hat{e}}$	$log|G_{1}\left|+log\right|Z_{p}|$	$log|G_{1}|$
Wu et al. [82]	$10T_{exp}$	$11T_{exp}+T_{\hat{e}}$	$5T_{exp}+2T_{\hat{e}}$	$2log|G_{1}|$	$2log|G_{1}\left|+log\right|G_{t}|$
Lu et al. [83]	$3T_{exp}$	$T_{exp}$	$T_{exp}$	$2log|G_{t}|$	$log|Z_{p}|$

**Table 15:** Notations for PEKS-CLE schemes.

Notation	Description
$T_{exp}$	The time of a modular exponentiation
$T_{\hat{e}}$	The time of a bilinear pairing
$|G_{1}|$	The number of elements in $G_{1}$
$|G_{t}|$	The number of elements in $G_{t}$
$|Z_{p}|$	The number of elements in $Z_{p}$

**Table 16:** Comparison of several PEKS-PRE schemes.

Scheme	Search Functionality	Security				Attack Model	
		Definition	Assumption	ROM	SCF	OKG	CA
Yau et al. [86]-I	Single	IND-CKA	BDH	✓			
Yau et al. [86]-II	Single	IND-CKA	BDH	✓	✓		
Wang et al. [88]	Conjunctive	wIND-CCA	q-BDHI	✓			
Guo et al. [89]	Verifiable	IND-CKA	QDBDH,DBDH,HDH		✓	✓	
Yang et al. [90]	Conjunctive	IND-CKA	DBDH,DDH		✓	✓	
Chen et al. [95]	Single	IND-CKA	q-BDHE	✓			✓

BDH refers to Bilinear Diffie–Hellman assumption. DBDH refers to Decisional Bilinear Diffie–Hellman assumption. q-BDHI refers to q-Bilinear Diffie–Hellman Inversion assumption. QDBDH refers to Quotient Decisional Bilinear Diffie–Hellman assumption. HDH refers to Hash Diffie–Hellman assumption. DDH refers to Decisional Diffie–Hellman assumption. q-BDHE refers to q-parallel Bilinear Diffie–Hellman Exponent assumption. CA denotes Collusion Attack, namely the proxy colluded with the delegate. We write wIND-CCA for weakly IND-CCA(chosen ciphertext attack).

**Table 17:** Efficiency of the compared PEKS-PRE schemes.

Scheme	Computation Cost			Communication Cost	
	Encrypt	Trapdoor	Test	Ciphertext Size	Trapdoor Size
Yau et al. [86]-I	$2T_{exp}+T_{\hat{e}}$	$T_{exp}$	$T_{\hat{e}}$	$log|G_{1}\left|+|\lambda \right|$	$log|G_{1}|$
Yau et al. [86]-II	$3T_{exp}+T_{\hat{e}}$	$3T_{exp}$	$2T_{exp}+T_{\hat{e}}$	$log|G_{1}\left|+|\lambda \right|$	$2log|G_{1}|$
Wang et al. [88]	$\left(4l+3\right)T_{exp}$	$T_{exp}$	$2T_{\hat{e}}$	$\left(2l+4\right)log|G_{1}|$	$3log|G_{1}|$
Guo et al. [89]	$3T_{exp}+T_{\hat{e}}$	$3T_{exp}$	$2T_{exp}+T_{\hat{e}}$	$log|G_{1}\left|+log\right|G_{t}|$	$2log|G_{1}|$
Yang et al. [90]	$\left(l+4\right)T_{exp}$	$\left(l+1\right)T_{exp}$	$\left(l+2\right)T_{\hat{e}}$	$\left(l+3\right)log|G_{1}|$	$\left(l+3\right)log|G_{1}|$
Chen et al. [95]	$\left(2N+4\right)T_{exp}$	$\left(S+4\right)T_{exp}$	$NT_{exp}+\left(2N+1\right)T_{\hat{e}}$	$\left(N+3\right)log|G_{1}|$	$\left(S+2\right)log|G_{1}|$

**Table 18:** Notations for PEKS-PRE schemes.

Notation	Description
$T_{exp}$	The time of a modular exponentiation
$T_{\hat{e}}$	The time of a bilinear pairing
$|G_{1}|$	The number of elements in $G_{1}$
$|G_{t}|$	The number of elements in $G_{t}$
$|Z_{p}|$	The number of elements in $Z_{p}$
*l*	The number of the keywords
|λ|	The bit length of security parameter
*S*	The number of a data user’s attribute
*N*	The number of attributes that are involved in a data owner’s access control policy

## Abbreviations

The following abbreviations are used in this manuscript:

PEKS	Public key encryption with keyword search
PEKS-PKI	Public key encryption with keyword search based on public key infrastructure
PEKS-IBE	Public key encryption with keyword search based on identity-based encryption
PEKS-ABE	Public key encryption with keyword search based on attribute-based encryption
PEKS-PE	Public key encryption with keyword search based on predicate encryption
PEKS-CLE	Public key encryption with keyword search based on certificateless encryption
PEKS-PRE	Public key encryption with keyword search supporting proxy re-encryption
ROM	Random oracle model
SCF	Secure channel free
OKG	Outside keyword guessing attack
IKG	Inside keyword guessing attack
FI	File injection attack
